# Physiological and Molecular Analysis Reveals the Differences of Photosynthesis between Colored and Green Leaf Poplars

**DOI:** 10.3390/ijms22168982

**Published:** 2021-08-20

**Authors:** Tao Wang, Lingyu Li, Guanghao Cheng, Xiaochun Shu, Ning Wang, Fengjiao Zhang, Weibing Zhuang, Zhong Wang

**Affiliations:** 1 Jiangsu Key Laboratory for the Research and Utilization of Plant Resources, Institute of Botany, Jiangsu Province and Chinese Academy of Sciences (Nanjing Botanical Garden Mem. Sun Yat-Sen), Nanjing 210014, China; johnwt@cnbg.net (T.W.); 17863656619@163.com (L.L.); chengguanghao520@163.com (G.C.); sxc@cnbg.net (X.S.); wangning813@njau.edu.cn (N.W.); fengjiao@cnbg.net (F.Z.); 2 Co-Innovation Center for Sustainable Forestry in Southern China, College of Biology and the Environment, Nanjing Forestry University, Nanjing 210037, China

**Keywords:** colored leaf poplar, photosynthesis, light and CO_2_ response curves, chlorophyll *a* fluorescence (OJIP), gene expression

## Abstract

Leaf coloration changes evoke different photosynthetic responses among different poplar cultivars. The aim of this study is to investigate the photosynthetic difference between a red leaf cultivar (ZHP) and a green leaf (L2025) cultivar of *Populus deltoides*. In this study, ‘ZHP’ exhibited wide ranges and huge potential for absorption and utilization of light energy and CO_2_ concentration which were similar to those in ‘L2025’ and even showed a stronger absorption for weak light. However, with the increasing light intensity and CO_2_ concentration, the photosynthetic capacity in both ‘L2025’ and ‘ZHP’ was gradually restricted, and the net photosynthetic rate (Pn) in ‘ZHP’ was significantly lower than that in ‘L2025’under high light or high CO_2_ conditions, which was mainly attributed to stomatal regulation and different photosynthetic efficiency (including the light energy utilization efficiency and photosynthetic CO_2_ assimilation efficiency) in these two poplars. Moreover, the higher anthocyanin content in ‘ZHP’ than that in ‘L2025’ was considered to be closely related to the decreased photosynthetic efficiency in ‘ZHP’. According to the results from the JIP-test, the capture efficiency of the reaction center for light energy in ‘L2025’ was significantly higher than that in ‘ZHP’. Interestingly, the higher levels of light quantum caused relatively higher accumulation of Q_A_^-^ in ‘L2025’, which blocked the electron transport and weakened the photosystem II (PSII) performance as compared with ‘ZHP’; however, the decreased capture of light quantum also could not promote the utilization of light energy, which was the key to the low photosynthetic efficiency in ‘ZHP’. The differential expressions of a series of photosynthesis-related genes further promoted these specific photosynthetic processes between ‘L2025’ and ‘ZHP’.

## 1. Introduction

With the rapid development of social economy, colored leaf plants are increasingly popular, which have been widely used in road greening, courtyard, and garden embellishment [1]. However, the different mechanisms of leaf coloration also change the physiological and biochemical adaptabilities in plants, especially the photosynthesis, which is often reduced in colored leaf plants to achieve special ecological functions, such as mimic defense and pollination [2]. The low photosynthetic capacity restricts the growth and development of colored leaf plants, and the restriction will be further aggravated under adversity stress [3,4], which is considered an important factor, limiting their geographically extensive promotion and application.

Photosynthesis is essential for plant growth and development, which incorporates numerous components, including CO_2_ assimilation pathways, photosynthetic photosystems, and the electron transport system [5,6]. The photosynthetic efficiency in colored leaf plants was mostly lower than that in green leaf plants, which was closely related to the decrease in chlorophyll (Chl) content, damage of photosystem II (PSII), and reduction in content and activity of enzymes related to photosynthetic CO_2_ assimilation in the former [3,7]. However, colored leaf plants are extremely sensitive to the external light environments, which evoke unpredictable effects on photosynthesis. For example, the photosynthetic efficiency and increased growth rate of red alga (*Pyropia haitanensis*) benefitted from light spectrums such as blue, green, and fluorescent tubes light, whereas red light has disadvantageous effects [8]. The acclimation of plants to different light environments induces biochemical responses associated with the remarkable plasticity of phenylpropanoid metabolism [9], which provides the possibility for genetic manipulation to improve the photosynthetic efficiency of color leaf plants. In recent years, several leaf color-related genes promoting high photosynthetic efficiency have also been reported, such as *Ygl7* in *Oryza sativa* L. ssp. *indica* [10] and *Ygl1* in *Setaria italic* [11]. Since the photosynthesis has a certain genetic stability in plants, this encourages an investigation of photosynthetic characteristics in colored leaf plants and a breeding of colored leaf varieties with high photosynthetic efficiency [12].

PSII is a multi-subunit pigment–protein complex embedded in the thylakoid membrane of oxygen-evolving phototrophs that supports light-driven oxidation of water to molecular oxygen and plastoquinone (PQ) reduction [13]. It has been described as the most important component affecting the photosynthesis of colored leaf plants. For most leaf color mutants, PSII activity is lower than that of normal green leaves, which have been proved in *Lagerstroemia indica* [14], maize [15], Chinese cabbage [16], and rice [17]. However, research on the plants with red or purple leaves was inconsistent, because the huge accumulation of foliar anthocyanins also plays an extremely critical role in the protection of photosynthetic apparatus from potentially damaging effects of supernumerary photons and reactive oxygen species [18,19], which even promoted the PSII photochemical efficiency under certain conditions [20]. Whether the formation of anthocyanins in colored-leaf plants participates in the photosynthetic responses is still controversial. Recently, the fast Chl *a* fluorescence induction (OJIP) curves have been successfully used in numerous studies to monitor the PSII performance, which is easy, fast, non-invasive, and provides plenty of information about the photochemical changes of PSII under various environmental conditions [21,22,23]. Therefore, research on the photosynthesis based on OJIP curves is an irreplaceable approach to elucidate the effects of anthocyanins on the photosynthetic electron transport and PSII activity in colored leaf plants.

Moreover, leaf color mutants also induced differential expression of photosynthesis-related genes, including coding for chloroplast proteins and other regulatory proteins or enzymes controlling genes, such as PSII reaction center D1 and D2 protein (*PsbA* and *PsbD*) [24], oxygen-evolving enhancer protein (*PsbO* and *PsbP*) [25], plastocyanin (*PetE*) [26], ferredoxin (*PetF*) [27], ferredoxin-NADP^+^ reductase (*PetH*) [28], Rubisco (*rbcL*) [29], and a series of F-type H^+^/Na^+^-transporting ATPase subunit genes in different plant species. These genes are necessary for normal growth and development as well as responses to environmental changes in plants, and could be utilized for characterizing photosynthesis-sensitive colored leaf genotypes.

**Figure 1:**
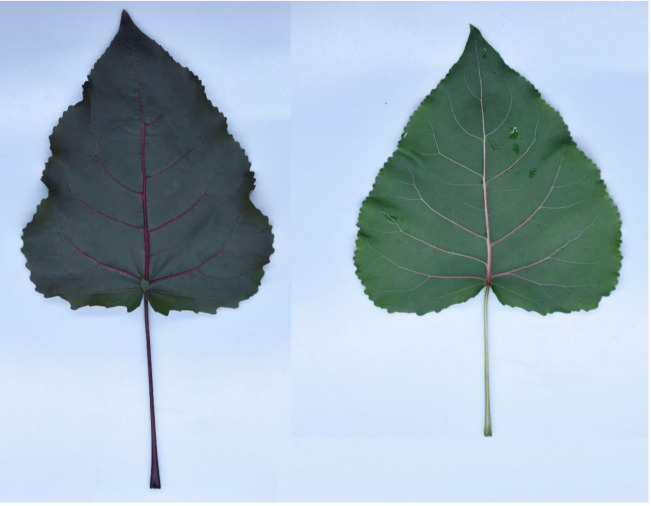
Typical appearances of ‘ZHP’ (**left**) and ‘L2025’ (**right**) seedling leaves.

Poplar is one of the most important fast-growing tree species in the northern hemisphere with huge economic, social, and ecological benefits [6]. The advantages of using members of the poplar genus (*Populus*) as models for the research on the tree physiological and molecular characteristics have been extensively reported [30,31,32]. *Populus deltoids* Linn. “2025” (L2025) is one of the most common poplar cultivars with rich resource and wide distribution for the protection and commercial forest in the plains and deserts of northern China. *Populus deltoids* “Zhonghong” (ZHP), originated from bud sports of the ‘L2025’, shows red leaves distinct from other poplar cultivars [33]. Typical appearances of ‘L2025’ and ‘ZHP’ leaves from seedlings with the same tree ages and branch were shown in Figure 1, which exhibited a similar leaf shape and size, but a dissimilar color. Due to the splendid ornamental values, ‘ZHP’ has been widely cultivated as a landscape tree in China and has attracted intense attention from breeders around the world as it was an ideal material for revealing the photosynthetic characteristics of colored leaf plants. Leaf pigment compositions potentially affect the photosynthetic capacity of ‘ZHP’, but information on photosynthetic mechanisms for ‘ZHP’ is limited in general, and previous studies have found little evidence of light reaction stages and molecular levels. In the present study, Chl *a* fluorescence combined with analysis of photosynthetic parameters and expression levels of photosynthesis-related genes were used to comprehensively investigate the differences of photosynthetic efficiency and light reaction activity between ‘L2025’ and ‘ZHP’ leaves. The results of this study will provide new insights into the photosynthetic mechanism for colored leaf plants. In addition, these results will provide scientific reference for the cultivation management and application of colored leaf poplars.

## 2. Results and Discussion

### 2.1. Changes in Chl, Carotenoid and Anthocyanin Contents between ‘L2025’ and ‘ZHP’ Leaves

Chl is the most important plant pigment as it plays a critical role in absorbing and transmitting light quantum [34]. Carotenoid is also crucial for the assembly of photosystems and light-harvesting chlorophyll–protein complexes (LHC). As shown in Table 1, the total Chl, Chl *a* and carotenoid contents showed no significant differences between ‘L2025’ and ‘ZHP’, which indicated consistent absorption and utilization of light energy and stable Chl synthesis in these two poplar leaves [35]. Chl *b* is favorable for the harvesting of dominant short-wavelength blue violet light in diffused light [36]. The Chl *b* content in ‘ZHP’ was 1.14 times higher than that in ‘L2025’ and the difference was significant. This indicated that ‘ZHP’ possessed greater ability in absorbing and utilizing weak light. The significant lower ratio of Chl *a*/Chl *b* in ‘ZHP’ than that in ‘L2025’ further indicated a stronger shade tolerance of ‘ZHP’. All these features contributed to the efficient interception and absorption of light for use in carbon gain. Moreover, the anthocyanin content of ‘ZHP’ was significantly higher than that of ‘L2025’, which could be one of the main factors for the leaf coloration in ‘ZHP’. Anthocyanins contributed to leaf photoprotection throughout the leaf development, and were tightly coordinated with carotenoids [37]. This process was believed to potentially affect the photosynthetic efficiency of ‘ZHP’.

### 2.2. Changes in Light Response Curves and CO_2_ Response Curves between ‘L2025’ and ‘ZHP’ Leaves

**Figure 2:**
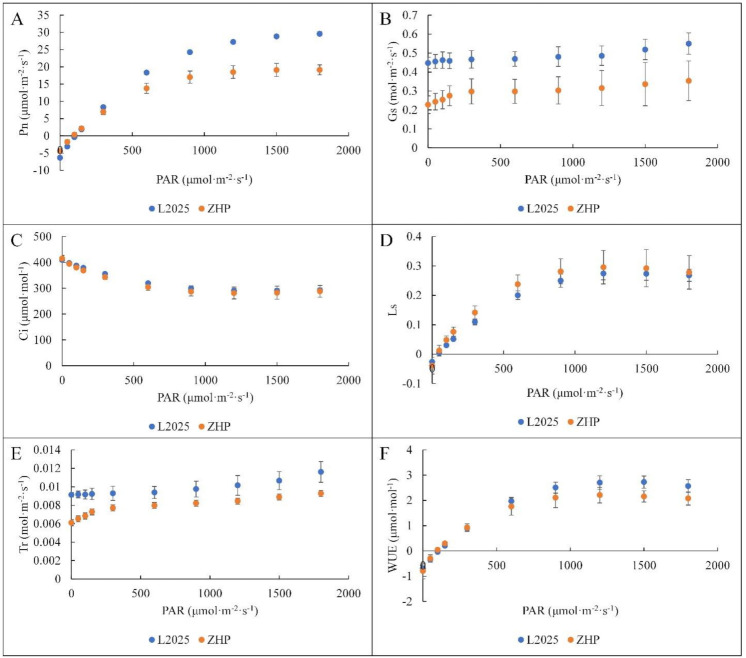
Changes in photosynthetically active radiation (PAR) responses from the leaves between ‘L2025’ and ‘ZHP’: (**A**) net photosynthetic rate (Pn). (**B**) stomatal conductance (Gs). (**C**) intercellular CO_2_ concentration (Ci). (**D**) stomatal limitation (Ls). (**E**) transpiration rate (Tr). (**F**) water use efficiency (WUE). Data are means ± SE (*n* = 3).

Light intensity is one of the most important environmental factors affecting photosynthetic processes in plants, including the energy supply for the formation of assimilatory ability, activation of key enzymes involved in photosynthesis, as well as the formation of Chl and the development of chloroplast [36,38,39,40]. In this study, net photosynthetic rate (Pn) showed a similar trend in ‘L2025’ and ‘ZHP’ with increasing photosynthetically active radiation (PAR), which increased rapidly as PAR increased to 300 μmol·m^−2^·s^−1^, and then increased slowly. The Pn values of ‘ZHP’ were significantly lower than those of ‘L2025’ after PAR reached 600 μmol·m^−2^·s^−1^, which increased to a maximum (19.13 μmol·m^−2^·s^−1^) at 1800 μmol·m^−2^·s^−1^, while that of ‘L2025’ was 29.64 μmol·m^−2^·s^−1^ (Figure 2A). The trend of Pn was similar to that of stomatal limitation (Ls), and was opposite to that of intercellular CO_2_ concentration (Ci), respectively, but no significant differences in the Ci and Ls values were found in ‘L2025’ and ‘ZHP’ (Figure 2C,D). The stomatal conductance (Gs) did not vary significantly with increasing PAR, while the Gs values in ‘L2025’ were significantly higher than those in ‘ZHP’ (Figure 2B). According to the judgment basis proposed by Farquhar and Sharkey [41], the stomatal limitation was considered a main factor resulting in the restriction of Pn with increasing light intensity in ‘L2025’ and ‘ZHP’. The stomatal behavior is considered an important strategy for plants to respond to changes in external environmental conditions [42]. The trend of transpiration rate (Tr) was similar to that of Gs in ‘L2025’ and ‘ZHP’ with increasing light intensity (Figure 2E), indicating a strict regulation of the stomatal behavior on the maintaining of water homeostasis [23,43]. Liu et al. [44] reported that the plants can reduce Tr as much as possible without significantly affecting Pn to reach the highest water use efficiency (WUE), which is considered an important strategy against environmental stress. As compared to ‘L2025’, ‘ZHP’ always maintained lower Tr levels; however, the WUE values of ‘L2025’ were mostly higher than those of ‘ZHP’ and the difference became more obvious as the light intensity increased (Figure 2F). The lower Tr in ‘ZHP’ did not improve the CO_2_ uptake, which indicated that the significant difference of Pn between ‘L2025’ and ‘ZHP’ was mainly attributed to nonstomatal factors [45]. It has been reported that anthocyanins are considered apposite light filters for photosynthetic organs, and that the extent of photoprotection depends strongly on the light conditions [20]. In this study, the anthocyanin content in ‘ZHP’ was significantly higher than that in ‘L2025’, which could gradually reduce the absorption of light energy by Chl in ‘ZHP’ with the increasing light intensity, resulting in decreased photosynthetic rates and minor changes in photosynthetic structures. This also explained the significant differences of Gs between ‘ZHP’ and ‘L2025’. Photoprotection by anthocyanins provides a functional advantage in the responses of sensitive photosynthetic apparatus to high light stress. This is also the basis for evaluating growth suitability in ‘L2025’ and ‘ZHP’.

**Figure 3:**
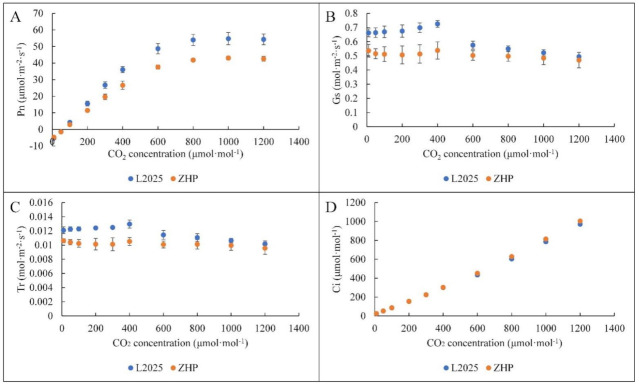
Changes in CO_2_ concentration responses from the leaves between ‘L2025’ and ‘ZHP’: (**A**) net photosynthetic rate (Pn). (**B**) stomatal conductance (Gs). (**C**) transpiration rate (Tr). (**D**) intercellular CO_2_ concentration. Data are means ± SE (*n* = 3).

The photosynthesis of plants is not only affected by light intensity, but also by the CO_2_ concentration [46,47]. In this study, Pn in both ‘L2025’ and ‘ZHP’ showed a trend of a rapid increase as CO_2_ concentration increased to 400 μmol·mol^−1^, and a slow increase thereafter, with the maximum values appeared at CO_2_ concentration of 1000 μmol·mol^−1^. The Pn values of ‘L2025’ were significantly higher than those of ‘ZHP’ after CO_2_ concentration reached 200 μmol·mol^−1^ (Figure 3A). Moreover, with increasing CO_2_ concentration, Gs and Tr in ‘L2025’ showed a consistent trend of a slow increase at first then a rapid decrease as CO_2_ concentration reached 400 μmol·mol^−1^, while that in ‘ZHP’ was fluctuating. The Gs and Tr values in ‘L2025’ were significantly higher than those in ‘ZHP’ as CO_2_ concentration increased to 400 μmol·mol^−1^, but the difference gradually decreased thereafter (Figure 3B,C). Elevated CO_2_ concentration affected both the carbon and the water dynamics in ‘L2025’ and ‘ZHP’, which further reflected the regulation of the sensitive stomatal behavior and the resulting impact on photosynthesis, as well as the genotypic differences in the photosynthetic responses. Interestingly, Ci increased rapidly with the increase in CO_2_ concentration and showed no significant differences between ‘L2025’ and ‘ZHP’ (Figure 3D), indicating an increasing proportion of non-stomatal factors. Higher CO_2_ concentrations can stimulate Pn by increasing CO_2_ substrate availability for rubisco and suppressing photorespiration [48]. However, as Ci increases, photosynthesis becomes limited by the ability to regenerate RuBP and produce starch and sucrose, which is less CO_2_-sensitive than rubisco carboxylation [49]. Therefore, rising CO_2_ should have the greatest effects on plant carbon uptake in conditions where Ci is low [50]. For ‘ZHP’, if lower Gs is paired with more leaf area in a high CO_2_ environment, these responses can offset the higher evaporative demand of a warmer climate in the future and thereby promoting the productivity and yield [51,52].

### 2.3. Model Fitting of Pn-PAR Response Curves and Pn-CO_2_ Concentration Response Curves in ‘L2025’ and ‘ZHP’ Seedling Leaves

**Figure 4:**
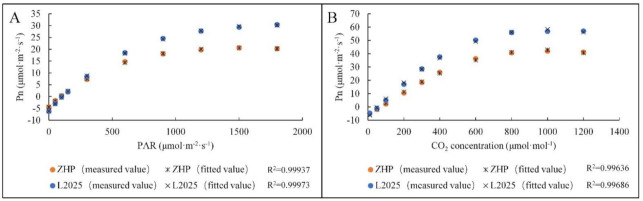
Model fitting of (**A**) Pn-PAR response curves and (**B**) Pn-CO_2_ concentration response curves from leaves between ‘L2025’ and ‘ZHP’.

A variety of models describing the Pn-PAR and Pn-CO_2_ concentration responses have been established for plants, including non-rectangular hyperbola, rectangular hyperbola, and exponential-based functions. However, these models lack widespread applicability as they do not consider the underlying biophysical and biochemical processes of photosynthesis [53]. Recently, a modified rectangular hyperbolic model was proposed by Ye and Yu [54], which specifically described light-harvesting characteristics and associated biophysical parameters of photosynthetic pigment molecules, and thus steadily reproduced the light response trends of both electron transport and CO_2_ uptake [55,56,57]. Moreover, this model also showed a better fitting effect on Pn-CO_2_ concentration responses in most plants [58]. In this study, the modified rectangular hyperbolic model was established based on ‘L2025’ and ‘ZHP’ to fit the light response and CO_2_ response processes. The representative line charts describing the Pn-PAR and Pn-CO_2_ concentration responses in ‘L2025’ and ‘ZHP’ are shown in Figure 4A,B, respectively. Interestingly, the measured values in both Pn-PAR and Pn-CO_2_ concentration responses from ‘L2025’ and ‘ZHP’ were highly consistent with the fitted values, and the determination coefficient (R^2^) was above 0.99, which indicated that this model fitted very well. Since the model introduces new corrected coefficients, which could deal with the photoinhibition of plants under high light conditions, the photosynthetic characteristic parameters for Pn-PAR responses, especially the A_max_ and LSP, could be fitted more precisely [53,59]. Correspondingly, this model also optimized the parameters CSP, CE, and Rp when fitting Pn-CO_2_ concentration responses [58], which is conductive to accurate assessment of poplar photosynthetic characteristics.

### 2.4. Changes in Photosynthetic Characteristic Parameters between ‘L2025’ and ‘ZHP’ Leaves

LCP and LSP reflect the adaptability of plants to light conditions. As shown in Table 2, the LCP and LSP values of ‘ZHP’ were lower than those of ‘L2025’, but the difference was not significant, which indicated that ‘ZHP’ has a wide range of light adaptability that was similar to ‘L2025’. However, the A_max_ and AQE values in ‘L2025’ showed 1.53 times and 1.19 times than those in ‘ZHP’, respectively, and the difference was significant. A_max_ represents the maximum photosynthetic capacity of leaves, and also reflects the risk of photoinhibition in strong light [60], while AQE is a powerful tool for assessing the utilization efficiency of light energy in weak light [59]. This indicated that the photosynthetic efficiency in ‘L2025’ was always higher than that in ‘ZHP’ under different light conditions. Moreover, Rd plays an important role in carbon sequestration for plants. The lower Rd in ‘ZHP’ could reduce consumption of photosynthetic assimilates, thereby keeping the stable accumulation of dry matter under the condition of lower Pn as compared to ‘L2025’. In general, shade-tolerant plants tend to have low photosynthetic capacity and LCP, and relatively high AQE, as shade leaves had a relatively high Chl *b* content and high levels of LHC to receive as much light as possible in low light levels, which may help maintain a positive carbon balance [61]. However, this is not completely consistent with the situation presented by ‘ZHP’. It has been reported that all red anthocyanins absorb green light, which induced fewer green photons reaching chloroplasts in more red leaves than in green leaves. This process resulted in a “shade acclimation syndrome” in anthocyanic morphs that could explain the lack of some traits typical of normal shade leaves [37]. It further implied potential effects of anthocyanins on the photosynthetic efficiency in ‘ZHP’.

As shown in Table 3, no significant differences in the CCP and CSP values were found between ‘L2025’ and ‘ZHP’, indicating an extensive utilization of CO_2_ concentration ranges in ‘ZHP’ that was similar to ‘L2025’. Therefore, higher CO_2_ concentration may be conducive to the rapid growth of ‘ZHP’ by stimulating the photosynthetic capacity. However, CE of ‘L2025’ was significantly higher than that of ‘ZHP’, which indicated that ‘L2025’ can synthesize more photosynthetic products than ‘ZHP’ to make up for the consumption by Rd. This may be attributed to the differences of rubisco activity between ‘L2025’ and ‘ZHP’. Moreover, the Rp value in ‘L2025’ showed 1.22 times than that in ‘ZHP’ with significant difference, which was considered an important mechanism for ‘L2025’ to protect photosynthetic apparatus from environmental stress by dissipating excess light energy [62]. The response mechanisms of cyanic and acyanic leaves of *Ocimum basilicum* suffering from an excess of solar irradiance have been reported by Torre et al. [63], who found that the energy dissipation process mediated by epidermal cyanic filter showed more advantages. Since the cyanic leaves display a greater capacity to absorb over the UV region of the solar spectrum, it also implied a wide adaptability of ‘ZHP’ in response to environmental stress.

### 2.5. Changes in OJIP Curves between ‘L2025’ and ‘ZHP’ Leaves

**Figure 5:**
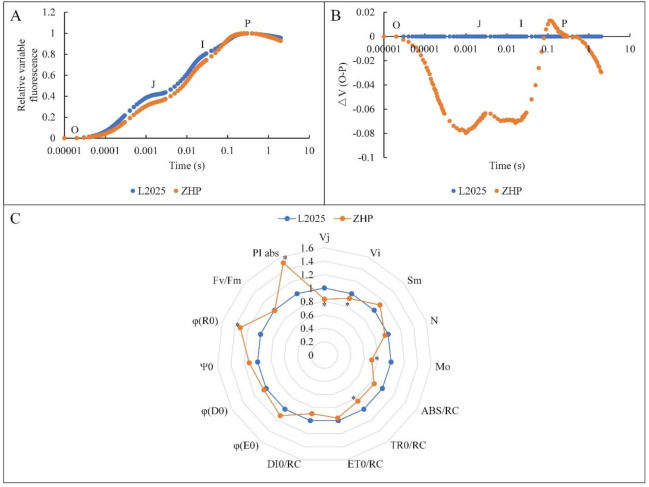
Changes in (**A**) standardized fluorescence intensity, (**B**) standardized variable fluorescence difference (ΔV), and (**C**) Chl *a* fluorescence parameter from leaves between ‘L2025’ and ‘ZHP’. Data are means ± SE (*n* = 4). Values followed by * indicate significant difference according to Student’s *t*-test (*p* < 0.05).

To further investigate anthocyanin-mediated PSII primary photochemical reaction and changes in the structure and function of photosynthetic apparatus, Chl *a* fluorescence method was applied between ‘L2025’ and ‘ZHP’ leaves. In this study, the fluorescence kinetics in both ‘ZHP’ and ‘L2025’ exhibited typical O-J-I-P polyphasic transient curves. The curve shapes were similar and the time to reach the P-step was basically the same (Figure 5A). The standardized relative variable fluorescence kinetics further showed the differences of ‘ZHP’ and ‘L2025’ mainly appearing between K-step and I-step, with the maxima near the J-step; and the values in ‘L2025’ were higher than those in ‘ZHP’ during the process (Figure 5B). K-step is related to the stability of the oxygen-evolving complex (OEC) [64]. The difference of K-step between ‘ZHP’ and ‘L2025’ implied a stronger ability of the donor side by ‘ZHP’ to supply electrons downstream, which may benefit from the structural and functional stability of photosynthetic apparatus mediated by anthocyanins, as anthocyanins could significantly alleviate the direct or indirect oxidative damage of the photosynthetic apparatus and DNA in plants in high light exposure [65]. The JI phase is suggested to mainly reflect the reduction in the intersystem electron carriers. Therefore, the higher levels of J-step and I-step in ‘L2025’ than those in ‘ZHP’ indicated different states of electron transport from Q_A_ to Q_B_, which was attributed to the heterogeneity of the PQ pool.

The JIP-test, based on the energy fluxes in biofilm, provides a convenient tool for the quantitative analysis of photosynthetic behavior from the absorption of light by PSII antenna to the reduction in the end electron acceptors driven by PSI [21]. A series of basic fluorescence parameters with important physiological significance were selected for a radar chart using ‘L2025’ as a reference (Figure 5B). In this study, the trapped excitation flux (leading to Q_A_ reduction) per reaction center (TR_0_/RC) in ‘L2025’ was significantly higher than that in ‘ZHP’, while this specific energy fluxes (per reaction center) for absorption (ABS/RC), electron transport (ET_0_/RC), and dissipation at the level of the antenna chlorophyll (DI_0_/RC) in ‘L2025’ were higher than those in ‘ZHP’, but with insignificant difference. These combined results showed a relatively activated reaction center in ‘L2025’ with stronger capacity of light harvesting as compared to ‘ZHP’, which also proved the restriction of anthocyanins on the capture of light quantum by LHC in ‘ZHP’ indirectly. However, the approximated initial slope of the fluorescence transient (M_o_) and the relative variable fluorescence intensities at the J-step (Vj) and I-step (Vi) of ‘L2025’ were significantly higher than those of ‘ZHP’, implying a higher reduction rate of Q_A_, accumulation of Q_A_^-^, and an energy dissipation ratio as electron transport to Q_B_ in ‘L2025’ [66]. Strasser et al. [67] indicated that, as the electron transport downstream of Q_A_^-^ was suppressed, M_o_ gradually reached the maxima. Combined with the Vi values, it further reflected a relative block of electron transport from Q_A_ to Q_B_ in ‘L2025’ as compared to ‘ZHP’. There is little difference in ‘L2025’ and ‘ZHP’ for maximum quantum yield of primary PSII photochemistry (φP_0_), quantum yield of the electron transport flux from Q_A_ to Q_B_ (φE_0_), and quantum yield of energy dissipation (φD_0_), and so there were times when Q_A_ reduced to Q_A_^−^ (N), the pool size of electron carriers per reaction center (S_m_), and the probability that a trapped exciton moves an electron into the electron transport chain beyond Q_A_^−^ (Ψ_0_), which indicated that the potential and activity of photosynthetic apparatus in ‘L2025’ and ‘ZHP’, were almost the same. However, more light quantum was captured by the reaction center of ‘L2025’, which eventually caused more electrons accumulated near Q_A_ of ‘L2025’ as the electron transport rate from Q_A_^−^ to Q_B_ was much slower than the reduction rate of Q_A_ [66]. This could be the key resulting in the accumulation of Q_A_^−^ and the block of electron transport in ‘L2025’. Moreover, the efficiency that an electron is transported from Q_B_ to the final electron acceptors of PSI (φR_0_) in ‘ZHP’ was significantly higher than that in ‘L2025’, further proving a stronger activity of electron transport chain in ‘ZHP’. It has been reported that Chl *a* fluorescence parameters differ between abiotic stress types, which allowed us to select some parameters as early indicators of a particular abiotic stress, such as the performance index (PI_abs_) and φD_0_ that were mostly sensitive to high light stress in rice seedlings [68]. In the present study, more trapped light quantum by the reaction center of ‘L2025’ mainly caused a decrease in PI_abs_ and an increase in Vj and Vi, which might be more suitable for characterizing the effect of high light stress on poplar species. The difference seems to be closely related to characteristics of the species itself. Moreover, PI_abs_ combines the individual effects of RC/ABS (the density of active reaction centers per chlorophyll absorption), φP_0_, and Ψ_0_ [69]. The high PI_abs_ level in ‘ZHP’ indicated that the performance of photosynthetic apparatus in ‘ZHP’ was prominent. Interestingly, the excellent electron transport chain in ‘ZHP’ was mainly from the decrease in the capture of light quantum by reaction center, which thereby could not promote the efficiency of absorption and utilization of light energy. However, the photosynthetic characteristics and the photoprotection by anthocyanins of ‘ZHP’ inversely promote the adaptability to adversity stress.

### 2.6. Changes in the Expression of Photosynthesis-Related Genes between ‘L2025’ and ‘ZHP’ Leaves

**Figure 6:**
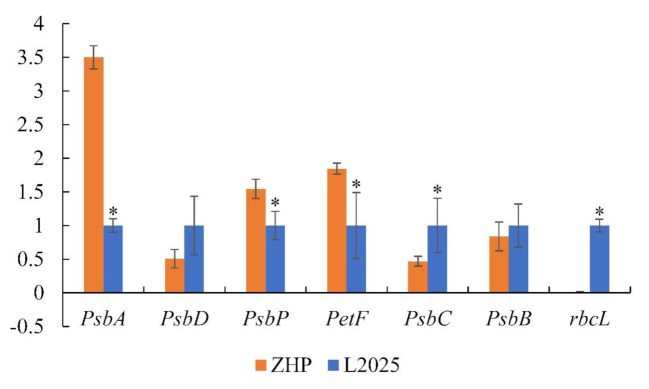
Expression analysis of selected photosynthesis-related genes from leaves between ‘L2025’ and ‘ZHP’ as determined by qPCR. Data are means ± SE (*n* = 3). Values of * indicate significant difference according to Student’s *t*-test (*p* < 0.05).

To analyze the genetic variation of photosynthesis between ‘L2025’ and ‘ZHP’ leaves, the expression level of selected photosynthesis-related genes was studied by using qRT-PCR methods. Among them, *PsbA*, *PsbP*, and *PetF* were more predominantly expressed in ‘ZHP’ than those in ‘L2025’, while that of *PsbC* and *rbcL* was opposite. *PsbD* and *PsbB* were lower expressed in ‘ZHP’ than those in ‘L2025’, but with no significant difference (Figure 6). The PSII reaction center is composed of a D1-D2 heterodimer, which binds chlorophyll, carotenoid, and PQ molecules for the light-dependent oxygen evolution and photophosphorylation-coupled linear electron flow [13,70,71]. The D1 protein is known to be rapidly degraded by PAR while the D2 protein is relatively stable [72]. Therefore, the increased expression of *PsbA* in ‘ZHP’ was an important supplement for the damaged D1 protein and was fairly vital for PSII recovery [73]. It also improved more activated water splitting systems and the downstream electron transport chain, which could be reflected by the highly expression of *PsbP* in ‘ZHP’. Moreover, the higher expression of *PetF* in ‘ZHP’ than in ‘L2025’ could explain the more efficient final electron acceptors in ‘ZHP’, which further emphasized the superiority of electron transfer chain in ‘ZHP’. CP43 and CP47 were the intrinsic transmembrane proteins located in the reaction center of PSII, which were used to couple the light harvesting antennas [74]. In the present study, the increased level of *PsbC* in ‘L2025’ conferred higher efficiency for light absorption and utilization. In addition, Rubisco could act as an oxygenase involved in catalyzing the first step of the plant photorespiration pathway and a carboxylase mediating CO_2_ assimilation [75]. The higher expression of *rbcL* gene in ‘L2025’ could provide a reasonable explanation for the higher Rp and CE values as compared to ‘ZHP’.

## 3. Materials and Methods

### 3.1. Plant Material and Growth Conditions

Two *Populus deltoids* cultivars, ‘ZHP’ with bright red leaves and wild-type ‘L2025’ with green leaves, were cultivated at the experimental field of Nanjing botanical garden Mem. Sun Yat-Sen, Nanjing China (32°3′ N, 118°49′ E). This area belongs to the subtropical humid monsoon climate zone, with an annual average temperature of 16.2 °C and an annual average precipitation of 1013 mm. The soil used in this field was yellow-brown soil. For the cultivating stage, individuals of ‘ZHP’ and ‘L2025’ at same ages were cultivated closely and under the same conditions, such as sunshine and water. Eight one-year-old seedlings of ‘ZHP’ and ‘L2025’ by cutting propagation with similar heights and growth conditions were selected, and six to eight fully expanded and healthy leaves from the third branch (from top to bottom) per plant were marked for the photosynthetic measurements in mid-May, 2020. Then, the leaves were collected for the measurements of Chl content and the expression levels of corresponding genes.

### 3.2. Measurement of Chl, Carotenoid and Anthocyanin Contents

The Chl and carotenoid contents in the leaves of ‘ZHP’ and ‘L2025’ were measured by an 80% acetone extraction method according to Huang et al. [61] with minor modifications. Briefly, fresh leaves (0.1g) were pulverized with distilled water and the homogenate was extracted with 10 mL of 80% acetone. The absorbance of the supernatant was measured at 665, 649, and 470 nm using a UV-2102PC/PCS ultraviolet spectrophotometer (UNICO, Shanghai, China). The Chl content was expressed as mg/g fresh weight (FW).

The total anthocyanin content in leaves of ‘ZHP’ and ‘L2025’ was measured based on the method described by Zhuang et al. [1]. About 1.0 g of fresh leaves was immersed into 10 mL of ethanol with 1% (*v/v*) HCl at 60 °C for 30 min. The mixture was centrifuged at 13,000× *g* for 5 min, and then the supernatant was obtained, which was used to measure the absorbance with a spectrophotometer at 530, 620, and 650 nm. The anthocyanin content was expressed as mg/g fresh weight (FW).

### 3.3. Measurements of Light Response Curves and CO_2_ Response Curves

Light response curves in leaves of ‘ZHP’ and ‘L2025’ were measured using an LI-6800 portable photosynthesis system (LI-COR, Lincoln, NE, USA) equipped with a multiphase flash fluorometer and chamber (6800-01F) on the same three sunny days from 08:30 to 11:30 h. The setting values of PAR was in turn 1800; 1500; 1200; 900; 600; 300; 150; 100; 50; 0 μmol·m^−2^·s^−1^ for 20 min per step, and an additional interval of 5 min per step for data collection. The leaf temperature (Tl) inside the sample chamber was at 25 °C and the relative humidity (RH) at 50%. A constant CO_2_ concentration of 400 μmol·mol^−1^ in the sample chamber (Ca) was provided with a CO_2_ injection system. Three marked leaves were selected from different individuals per cultivar and measured repeatedly. The measured parameters included Pn, Gs, Ci, Tr. In addition, Ls was calculated using the formula of Ls = 1 − Ci/Ca, and WUE was calculated as Pn/Tr.

CO_2_ response curves were determined after the measurements of light response curves with the LI-6800 portable photosynthesis system under the same conditions (Ca of 400 μmol·mol^−1^, Tl of 25 °C, and RH of 50%) inside the leaf chamber. Before measurements, the saturating light was set at 1200 μmol·m^−2^·s^−1^, and then the CO_2_ concentration was set following the order of 400; 300; 200; 100; 50; 10; 400; 400; 600; 800; 1000; 1200 μmol·mol^−1^. The leaves measured for the light response curves were also used in this measurement.

The resulting Pn-PAR curves and Pn-CO_2_ concentration curves were fitted by a modified rectangular hyperbolic model [53]. Parameter estimation was accomplished by an online tool (http://photosynthetic.sinaapp.com/calc.html, accessed on 12 May 2021). In the model for Pn-PAR curves, LCP is the light compensation point; LSP is the light saturation point; A_max_ is the maximum net photosynthetic rate; AQE is the apparent quantum efficiency; and Rd is the dark respiration rate. In addition, according to the model for Pn-CO_2_ concentration curves, CCP is the CO_2_ compensation point; CSP is the CO_2_ saturation point; CE is the carboxylation efficiency; and Rp is the photorespiration rate.

### 3.4. Measurement of Chl Fluorescence

The Chl *a* fluorescence transient was measured using a Handy PEA (Hansatech, UK), with the PEA probe fixing on the central position of the marked leaves from different individuals per cultivar. For each cultivar, measurements were repeated at least four times. The leaves were dark-adapted for 30 min before the measurement, and the data were analyzed by the JIP-test, according to the methods of Strasser et al. [67]. The JIP-test is used to quantitatively analyze and understand the OJIP transient, and to reveal the environmental effect on the structure, conformation, and function of the photosynthetic organisms. A typical JIP-test included three phases: O-J (0.05-5 ms), J-I (5-50 ms), and I-P (50-1000 ms), which provided a large amount of information about the donor side, the acceptor side, and the reaction center of PSII. The introduced basic fluorescence parameters are listed below: ABS/RC, average absorbed photon flux per PSII reaction center; TR_0_/RC, the specific energy fluxes per reaction center for trapping; ET_0_/RC, the specific energy fluxes per reaction center for electron transport; DI_0_/RC, the specific energy fluxes per reaction center for dissipation; Vj, relative variable fluorescence at the J-step; Vi, relative variable fluorescence at the I-step; S_m_, the normalized area (assumed proportional to the number of reduction and oxidation of one Q_A_^−^ molecule during the fast OJIP transient and therefore related to the number of electron carriers per electron transport chain); M_o_, the approximate value of the initial slope of relative variable Chl fluorescence curve V_t_ (for F_0_ = F_50μs_); N, the times Q_A_ was reduced to Q_A_^−^ in the time span from t_0_ to t_Fmax_; φP_0_, the maximum quantum yield of primary PSII photochemistry; φE_0_, the quantum yield for electron transport; φD_0_, the quantum yield (*t* = 0) of energy dissipation; φR_0_, the quantum yield for reduction in the end electron acceptors at the PSI acceptor side; Ψ_0_, the efficiency with which a trapped exciton moves an electron into the electron transport chain beyond Q_A_^−^; and PI_abs_, the performance index for energy conservation from photons absorbed by PSII antenna to the reduction in intersystem electron acceptors.

### 3.5. Measurement of qPCR

For gene expression determination, the samples were first stored at −80 °C and were prepared for RNA isolation and gene expression analysis. Total RNA was isolated from about 0.1 g of crushed leaves by a plant RNA kit (Huayueyang, Beijing, China) according to the manufacturer’s protocol. The first strand of cDNA was synthesized from 1 μg of total RNA using PrimeScript RT reagent Kit (Takara, Shiga, Japan). The primers for the corresponding genes were designed on primer 5, and actin2 was used as an internal control (Table 4). The qPCR was performed in Applied Biosystems 7500 Real-Time PCR System (Applied Biosystems, Waltham, MA, USA) with SYBR Green II PCR Master Mix (Takara, Shiga, Japan). The qPCR was carried out in a final volume of 20 μL, which contained 4 μL of cDNA, and the conditions were the following: initial denaturation at 95 °C for 30 s, 40 cycles of denaturation at 95 °C for 5 s, and annealing and extension at 60 °C for 34 s. A melting curve was obtained at 95 °C for 15 s and at 60 °C for 1 min followed by continuous heating. The analysis of the qPCR results was performed with the REST 2009 software.

### 3.6. Statistical Analysis

The experiments were replicated three times independently. The results were expressed as mean ± SE of at least three biological replicates. Statistical analysis was conducted using SPSS software version 19.0 and Microsoft Excel 2019. The graphs were produced using Microsoft Excel 2019 and Microsoft Visio 2019. Values followed by * in graphs and tables indicate significant differences between ‘ZHP’ and ‘L2025’ based on Student’s *t*-test (*p* < 0.05).

## 4. Conclusions

‘ZHP’, originated from bud sports of the ‘L2025’, shows specific leaf coloration and photosynthetic characteristics distinct from its wildtype. In this study, the increasing light intensity and CO_2_ concentration gradually restricted the photosynthetic capacity in ‘ZHP’ and ‘L2025’, and Pn values in ‘L2025’ were significantly higher than those in ‘ZHP’ under high light and high CO_2_ environments. The reason was mainly concerning the strict regulation of stomatal behavior for the balance of CO_2_ uptake and water loss as well as the different photosynthetic efficiency (including the utilization efficiency for solar energy and the efficiency of photosynthetic CO_2_ assimilation) in these two poplars, while the expression levels of *PsbC* and *rbcL* genes could further explain the difference of photosynthetic efficiency in ‘L2025’ and ‘ZHP’. According to the results from pigment contents and photosynthetic characteristic parameters, ‘ZHP’ showed no significant difference from ‘L2025’ in the ranges and potential of absorption and utilization for light and CO_2_ concentration, and even possessed a stronger ability to absorb weak light. However, the higher anthocyanin content in ‘ZHP’ than that in ‘L2025’ potentially restricted the capture of light quantum by reaction center of ‘ZHP’, which was unable to promote the utilization of light energy, and eventually resulted in the decrease in photosynthetic efficiency in ‘ZHP’. Interestingly, the decreased light quantum also reduced the risk of Q_A_^-^ accumulation in ‘ZHP’. The relative expression levels of *PsbA*, *PsbP*, and *PetF* genes increased to higher levels in ‘ZHP’ than in ‘L2025’, conferring a more reasonable and optimized electron transport system to the former one. This potentially provides evidence for a wide adaptability of ‘ZHP’ in response to environmental stress.

## Figures and Tables

**Table 1:** Chl, carotenoid and anthocyanin contents in leaves between ‘L2025’ and ‘ZHP’.

	Chl *a* (mg/g FW)	Chl *b* (mg/g FW)	Chl *a*/Chl *b*	Total Chl (mg/g FW)	Carotenoid (mg/g FW)	Anthocyanin (mg/g FW)
ZHP	1.622 ± 0.104	0.428 ± 0.037 *	3.799 ± 0.167 *	2.049 ± 0.137	0.389 ± 0.032	0.268 ± 0.030 *
2025	1.604 ± 0.047	0.375 ± 0.013	4.275 ± 0.103	1.980 ± 0.058	0.371 ± 0.011	0.016 ± 0.002

Data are means ± SE (*n* = 4). Values followed by * indicate significant difference within the same column according to Student’s *t*-test (*p* < 0.05).

**Table 2:** Photosynthetic characteristic parameters of light response curves in ‘L2025’ and ‘ZHP’ seedling leaves.

	AQE	Rd (μmol·m^−2^·s^−1^)	A_max_ (μmol·m^−2^·s^−1^)	LCP (μmol·m^−2^·s^−1^)	LSP (μmol·m^−2^·s^−1^)
ZHP	0.052 ± 0.001 *	4.377 ± 0.590 *	19.287 ± 1.705 *	92.833 ± 13.324	1579.150 ± 145.846
L2025	0.062 ± 0.001	6.262 ± 0.239	29.508 ± 0.577	109.719 ± 5.970	1743.047 ± 70.205

Data are means ± SE (*n* = 3). Values followed by * indicate significant difference within the same column according to Student’s *t*-test (*p* < 0.05).

**Table 3:** Photosynthetic characteristic parameters of CO_2_ response curves in ‘L2025’ and ‘ZHP’ seedling leaves.

	CE (μmol·m^−2^·s^−1^)	Rp (μmol·m^−2^·s^−1^)	CCP (μmol·mol^−1^)	CSP (μmol·mol ^−1^)
ZHP	0.106 ± 0.013 *	6.598 ± 0.509 *	65.046 ± 3.825	995.966 ± 20.032
L2025	0.142 ± 0.006	8.067 ± 0.291	59.064 ± 4.637	983.085 ± 16.474

Data are means ± SE (*n* = 3). Values followed by * indicate significant difference within the same column according to Student’s *t*-test (*p* < 0.05).

**Table 4:** Specific primers used in relative qPCR.

Gene Name	Accession	Description	Forward Primer (5′to 3′)	Reverse Primer (5′ to 3′)
*PsbA*	Potri.013G143200	photosystem II P680 reaction center D1 protein	CTTAGTTTCCGTCTGG GTAT	GCTCAGCCTGGAATA CAATC
*PsbD*	Potri.008G208600	photosystem II P680 reaction center D2 protein	ATTAGGTGGCTTGTG GACTT	GAGCCAACCAAACTG ACCT
*PsbP*	Potri.010G210000	photosystem II oxygen-evolving enhancer protein 2	TCATTGAGTTGGGCTT CC	ATTGAAGGTTGCCTCT GC
*PetF*	Potri.001G470700	ferredoxin	GCTGGTGCTTGCTCTT CAT	CAGCCTCTATCTGGTC TTC
*PsbC*	Potri.010G032700	photosystem II CP43 chlorophyll apoprotein	TATTCCCTGAGGAGTT TCTAC	ATAAGTTCATTGCTCC GACCC
*PsbB*	Potri.011G113900	photosystem II CP47 chlorophyll apoprotein	TGTTGAGTTCTATGGT GGTG	ATCGGATTTCAAAGT AGCAC
*rbcL*	Potri.012G062600	ribulose-1,5-bisphosphate carboxylase/oxygenase	GAACTTGTAGCCTCA TCCG	TACGGAATCATCTCC AAAG
*Actin2*	Potri.019G010400.1	Actin	GCCATCTCTCATCGG AATGGAA	AGGGCAGTGATTTCC TTGCTCA
